# The Impact of Marijuana on the Cardiovascular System: A Review of the Most Common Cardiovascular Events Associated with Marijuana Use

**DOI:** 10.3390/jcm9061925

**Published:** 2020-06-19

**Authors:** Zara Latif, Nadish Garg

**Affiliations:** 1 Department of Medicine, Beth Israel Deaconess Medical Center, Harvard Medical School, 330 Brookline Ave, Boston, MA 02215, USA; 2 Division of Cardiovascular Disease, Department of Medicine, University of Tennessee Health Science Center, 910 Madison Ave, Memphis, TN 38163, USA; ndgarg@gmail.com

**Keywords:** marijuana, cardiovascular disease, cannabis, myocardial infarction, arrythmias, Takotsubo cardiomyopathy, cannabis arteritis

## Abstract

With the expanded legalization of marijuana, its medical and recreational use have sharply increased over the past decade. A wide array of new forms of cannabis is available on the market today, and the potencies are ten times those of forms previously tested, meaning that the true impact of marijuana on the cardiovascular system remains unclear. Cannabis mainly exerts its effects via the sympathetic and parasympathetic nervous systems, with different doses affecting different cannabinoids receptors. Studies have shown that marijuana plays a role in thrombosis, inflammation, and atherosclerosis. Case reports have linked marijuana use to myocardial infarction, cardiac arrythmias, cardiomyopathies, stroke, and arteritis. Most patients are young, healthy men with no cardiovascular risk factors; however, the patient population is expected to change to include older individuals in the future. The widespread public perception of safety accompanying marijuana use has contributed to its increased use among the elderly, who are the most at risk population for acute cardiovascular events. In this review, we aim to provide a basic understanding of the physiological effects of marijuana on the cardiovascular system and to review the current literature regarding cardiovascular diseases linked to marijuana use in adults.

## 1. Introduction

Over the last ten years, there has been a dramatic increase in medical and recreational marijuana use. As of 2020, 33 states plus the District of Columbia have legalized marijuana in the United States, with the number of states doing so expected to increase later in the year. On a global scale, there were an estimated 188 million marijuana users in 2017, which is approximately 3.8% of the global population aged 15–64 years old [1]. In 2018, 11.8 million young adults in the United States reported marijuana use [2]. The upward trend continued in 2019 when Monitoring the Future Study reported that an estimated 15.2% of eighth graders in the U.S. admitted to marijuana use, and 34% of 10th graders were stated to have used marijuana in the past year. The rates among 12th graders were as high as 43%, which are similar to those among college students [3]. The widespread use of marijuana is not limited to young adults; it is also gaining popularity among the elderly as a method of treatment for chronic illnesses and to improve quality of sleep. According to a study done by Azofeifa between 2002 and 2014, there was a 455% increase in marijuana consumption among U.S. adults aged 55–64 and a 333% increase in those older than 64 years, the most at risk population for cardiovascular events [4].

With the growing popularity of vaping among teens, the vaping of marijuana has more than doubled in the past two years with a reported rate of almost 20% among 10th and 12th graders [5]. It was hypothesized in the past that vaporized cannabis is safer than smoked. This is due to the lack of carbon monoxide formation with heating compared to combustion. Data suggest that vaporized marijuana produces greater psychoactive effects and higher blood delta 9-tetrahydrocannabinol (THC) concentrations [6]. However, with new reports of vaping being related to lung injury, vaping marijuana may prove to be more damaging than was originally thought [7].

Today, cannabis is the most frequently used psychoactive substance after alcohol and tobacco [6]. Its widespread use has been accompanied by a public perception of safety among young adults and the elderly. In a 2014 survey done among 13,128 people aged 15–24 in Europe, cannabis was considered the least harmful drug and the easiest to obtain [1]. The risk of developing dependence on cannabis among those who have ever used the drug is estimated to be between 9% and 30% based on studies done in the U.S. [8].

Over the past twenty years, there has been a myriad of reports and studies regarding the adverse cardiovascular impact of marijuana. A multicenter study published in 2011 reported that cardiovascular disorders accounted for 9.5% of the total adverse events among patients with cannabis-related hospital admissions [9]. The French Addictovigilance Network noted an increase in cardiovascular complication rates in marijuana users from 1.1% in 2006 to 3.6% in 2010. The mortality rate resulting from these complications was as high as 25% [10]. Interestingly, data from U.S. National Vital Statistics for 1990–2014 showed that cardiovascular-related mortality rates in states with liberal rules for marijuana demonstrated an increase in cardiac mortality of 2.3% in men and 1.3% in women compared to states where marijuana use was not legal [11]. The authors report that the effect was mostly seen in older individuals who used cannabis for symptoms like chronic pain rather than a specific illness. Over the years, marijuana consumption has been linked to acute myocardial infarction, cardiac arrythmias, cardiomyopathies, stroke, arteritis, and sudden cardiac death [12].

In this paper, we will review the current literature regarding the impact of marijuana on cardiovascular health, starting with a basic understanding of the physiological effects of cannabis on the cardiovascular system.

## 2. Cannabis

There are multiple cannabis species, each with a differing amount of the two major active ingredients, delta 9-tetrahydrocannabinol (THC) and cannabidiol (CBD) [6]. THC is responsible for the euphoric effects of cannabis, while CBD is often marketed for its anti-inflammatory actions. The synthetic, THC-like dronabinol and nabilone are used as treatments for nausea and vomiting [13]. In 2018, the U.S. Food and Drug Administration approved an oral solution of CBD for the treatment of two forms of epilepsy, Lennox-Gastaut and Dravet syndrome [14]. Medical marijuana has also been used for the treatment of neuropathic pain associated with cancers and neurological disorders [6].

## 3. Physiological Effects of Marijuana

To understand the impact of marijuana on the cardiovascular system, it is imperative to understand the endocannabinoid system. The endocannabinoid system (ECS) is comprised of the endocannabinoids anandamide and 2-arachidonylglycerol (both of which are endogenous lipid mediators), their metabolic enzymes, and G-protein coupled cannabinoid receptor 1 (CB1R) plus G-protein coupled cannabinoid receptor 2 (CB2R) [15]. CB1R is the primary receptor that mediates the effects of marijuana. CB1R is present in the brain, heart, vascular smooth muscle, and peripheral nervous system [13]. Its extensive presence in the human body makes its activation wide-reaching and impacts multiple systems.

### 3.1. Molecular Mechanisms of G-Protein Coupled Cannabinoid Receptors

The molecular mechanism of CB1R and CB2R signaling involves signal transduction via the modulation of adenylyl cyclase (AC), mitogen activated protein kinases (MAPK), and nuclear factor kappa light chain enhancer of activated B cells (NF-kb) [16]. CB1R stimulation causes the activation of adenylyl cyclase inhibitor subunit of G-proteins (Gi/o), resulting in reduced cAMP formation [17]. This in turn causes the inhibition of N-type calcium channels and activation of G-protein coupled inwardly rectifying potassium channels (GIRK) [18]. CB1R activation has also been shown to activate MAPK signaling pathways including p38, extracellular signal regulated kinase 1/2 (ERK1/2), and c-Jun N-terminal kinase (JNK), which are involved in cell proliferation, cell cycle control, and cell death. In addition to MAPK signaling, CB1R can activate the phosphatidylinositol-3-kinase (PI3K)/protein kinase B (Akt) pathway, which is responsible for cell growth and survival. CB1R can also signal via G-protein-independent mechanisms by associating with ß-arrestin. ß-arrestin plays a critical role in GPCR desensitization where it binds to the receptor and initiates the internalization process [18]. Interestingly, intracellular CB1R does not translocate but it can increase intracellular calcium through the release of internal lysosomal calcium stores [19]. CB1R located in the mitochondria can decrease mitochondrial respiration and cAMP formation, affecting cellular energy metabolism [20]. The dynamic nature of CB1R signaling results in different outcomes of CB1R activation, leading to either cell death or cell survival depending on the environment. While CB1R signaling is heavily studied, the role of CB2R in the cardiovascular effects of ECS is not well known.

### 3.2. Effects of Marijuana on the Autonomic Nervous System

**Figure 1:**
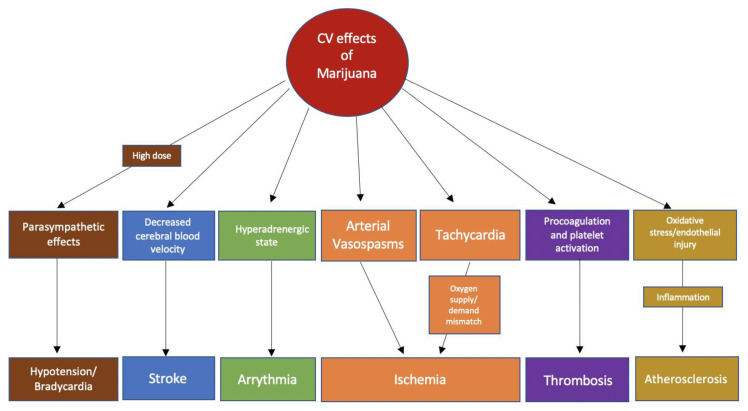
Possible pathophysiological mechanisms of the association of common cardiovascular events with marijuana use.

The ECS affects both the sympathetic and parasympathetic nervous systems [13]. Smoking marijuana results in immediate tachycardia and elevated supine blood pressure. In a study done by Beaconsfield et al., an immediate effect of smoking marijuana is a 20–100% increase in heart rate. The effects on heart rate can occur within 10 min of marijuana inhalation and last between 2 and 3 h [21]. The increase in heart rate is mediated mainly by CB1R activation. In a study where a CB1-selective receptor antagonist was used, the mean peak heart rate increase was diminished by 59% in healthy volunteers who smoked marijuana 2 h prior to dosing [22]. Similarly, the use of propranolol before marijuana attenuated the tachycardia, further supporting the idea that cannabis-induced tachycardia is attributable to sympathetic nervous system activation [23]. In addition, marijuana can inhibit cardiac parasympathetic innervation, which is supported by the finding that marijuana reduces vagal slowing during the Valsalva maneuver [24]. Lastly, it has been hypothesized that marijuana-induced vasodilation stimulates reflex tachycardia, which is likely to be an additional mechanism for cannabis-induced tachycardia [25]. On the other hand, in animal studies, the use of a cannabinoid agonist resulted in bradycardia, hypotension, and a reduction in noradrenaline concentrations, indicating parasympathetic stimulation by ECS [26]. The biphasic effect of cannabinoids is likely to be dose-dependent as indicated by some studies, where lower doses caused sympathetic stimulation and norepinephrine release while higher doses resulted in parasympathetic stimulation [27,28]. The main physiological effects of marijuana could be summarized as an increase in heart rate, enhanced sympathetic tone, increased catecholamine levels at lower doses, and bradycardia/hypotension at higher doses [12,19,27]. All of these effects could serve as pathophysiological mechanisms for common cardiovascular events linked to marijuana use (Figure 1).

Postural hypotension and dizziness have been seen with higher doses of marijuana. Healthy volunteers who experience orthostatic hypotension after smoking marijuana have also shown evidence of decreased cerebral blood velocity when assessed by transcranial Doppler. The decrease in cerebral blood velocity can increase the risk of ischemic strokes and increase the likelihood of falls resulting in injuries [29]. An increased risk for falls poses an important concern regarding the effects of marijuana in the elderly, especially with the recently increased use among that population.

### 3.3. Effects of Marijuana on Myocardial Oxygen Demand

Marijuana use has been indicated to adversely affect the myocardial oxygen supply and demand. The combination of an increased myocardial oxygen demand as a result of tachycardia and a decreased oxygen supply as a result of high carboxyhemoglobin levels creates a supply and demand mismatch, which can result in transient myocardial ischemia [30]. Smoking marijuana causes an increase in the amount of carboxyhemoglobin due to combustion, which in turn causes a decrease in oxygen supply. Following marijuana use, cardiac output increases by 4% to 9%, with an increase in cardiac work [30,31]. In addition, marijuana use increases the sinus rate, decreases exercise-related cardiac performance, and decreases systemic vascular resistance [30,32,33,34,35]. Cannabis has also been shown to decrease the end diastolic volume, stroke index, ejection fraction, and left ventricular ejection time [25]. All of these effects combined support the notion that the cardiac effects of marijuana are mainly chronotropic. Moreover, in an isolated human atrial myocardial tissue, CB1R activation decreases myocardial contractile performance [36].

### 3.4. Effect of Marijuana on Thrombosis

Multiple case reports have linked marijuana to thrombus formation, leading to acute myocardial infarction in young adults [15,37,38,39,40,41]. The pro-coagulant effect of marijuana is attributed to the presence of CB1R and CB2R on human platelets. This effect is also due to an increase in the surface expression of glycoprotein IIb-IIIa and P-selectin after THC exposure [42]. Activated CB1R has been implicated in promoting endothelial dysfunction, an important factor in atherosclerosis development. CB1R stimulation in endothelial cells activates pathways including MAPK. MAPK activation, in turn, triggers the release of mediators that interfere with normal vasodilation and result in the release of reactive oxygen species (ROS). A study showed that CB1R induced a concentration- and time-dependent stimulation of MAPK, which promoted cell death [43]. Combined, these effects lead to endothelial dysfunction and a pro-coagulant state. A CB1R antagonist mitigated and almost reversed these negative effects in some studies. [43,44].

### 3.5. Effect of Marijuana on the Inflammatory and Atherosclerotic Pathways

Marijuana has opposing effects on different receptors in relation to inflammation. CB1R activation has been implicated in the formation of oxidized low density lipoproteins (LDL) and the induction of an inflammatory response [24]. Studies focused on the effects of marijuana on CB2R have gained more attention in recent years. CB2R is expressed in immune tissue and hematopoietic cells; these receptors are upregulated in response to inflammation and tissue injury, linking them to anti-inflammatory effects [45]. Studies of human coronary artery endothelial cells have shown that CB2R agonists attenuate the pro-inflammatory processes triggered by CB1R activation [13]. The use of a CB2R agonist reduced oxidized LDL accumulation in macrophages along with other inflammatory markers implicated in atherosclerosis, suggesting that the activation of CB2R attenuates atherosclerosis. Thus, there has been significant interest in developing a selective CB2R agonist for the treatment of atherosclerosis [46]. There are multiple conflicting studies regarding atherosclerosis and marijuana use [44,47,48,49,50]. Current studies have, however, failed to demonstrate any direct impact of marijuana use on atherogenesis thus necessitating further investigation in this area.

### 3.6. Effect of Marijuana on Vascular Tissue

Cannabis has been shown to cause predominantly vasodilatory responses via the activation of transient receptor potential ankyrin type 1 (TRPA1) ion channels. However, vasoconstriction has been seen in coronary, cerebral, and peripheral arterial systems [6]. The contrasting effects of cannabinoids in different vascular tissues are attributed to the different endothelial vasodilator mechanisms in the tissues of interest. Myocardial blood flow, for example, has been shown to be inversely correlated with plasma levels of endocannabinoids [6]. In a study of an isolated mesenteric resistance vessel, THC was shown to inhibit the endothelium-dependent vasorelaxation. The same study demonstrated that THC produced vasorelaxation in aortic rings via enhanced nitric oxide availability, hydrogen peroxide production, and superoxide dismutase activity. The authors concluded that the effects of THC on endothelium-dependent vasorelaxation are dependent on the predominant endothelium-relaxing factor in a given artery, which subsequently results in a heterogenous effect of THC in different vascular beds [51]. Cannabinoids can also cause CB-receptor-independent vasodilatory effects by inhibiting voltage-gated calcium channels [16].

## 4. Pharmacokinetics

Cannabis comes in many forms and has different methods of use, with the most common being inhalation via smoking or vaporization. Inhalation is the fastest method of intoxication and results in a predictable bioavailability. Plasma THC levels are detectable within seconds to minutes of inhalation and reach a maximum at 15–20 min. In comparison, oral consumption is slower to take effect, inducing peak levels at 2–3 h, with less predictable bioavailability [13]. The route of administration influences the bioavailability and serum concentration of the active ingredient. In a study of healthy volunteers, the plasma THC concentrations and clinical effects were similar after smoking and IV injections, but ingestion induced a less predictable and delayed peak plasma THC concentration [6]. This is especially important now as edible forms of marijuana are gaining popularity among users. The erratic absorption and unpredictable bioavailability can create difficulties when deciding upon the best management strategy for these patients.

Cannabis metabolism results in the production of eighteen different classes of chemicals, including amino acids, fatty acids, hydrocarbons, terpenes, nitrogenous compounds, and carbohydrates [52]. Δ^9^-THC is metabolized in the liver by hydroxylation and oxidation, catalyzed mainly by the cytochrome P450 enzyme complex [53]. Phase I of hepatic metabolism is the oxidation reaction resulting in the psychoactive compound 11-hydroxy-THC (11-OH-THC), which is the primary metabolite and the result of THC hydroxylation by the CYP450 2C9 enzyme. The oxidation of 11-OH-THC produces the inactive metabolite 11-nor-9-carboxy-THC (THC-COOH), the compound detected in screening tests. THC-COOH and its glucuronide conjugate are the main end products of cannabis metabolism in humans [52]. THC-COOH is excreted in the urine as glucuronic acid conjugates, which increases its water solubility [53]. Over 65% of cannabis is excreted in the feces, and about 20% is excreted in the urine. Almost 90% of cannabis is excreted within 5 days as hydroxylated or carboxylated metabolites. ∆^9^-THC is extremely lipid-soluble, and that leads to tubular reabsorption, resulting in low renal excretion [53]. The extrahepatic metabolism of THC also occurs, with the side-chain hydroxylation of THC being the most prominent mechanism in the lungs. CBD metabolism is similar to that of THC with side-chain oxidation; the only significant difference in metabolism is that a large proportion of CBD is excreted in the feces unchanged [52].

## 5. Tolerance

Tolerance to the cardiovascular effects of marijuana develops rapidly and is lost rapidly when marijuana use is stopped. Multiple studies have demonstrated that after a few days of repeated marijuana exposure, the increase in heart rate and supine blood pressure are attenuated and then lost [24]. By the end of a 20-day trial of oral THC being given to healthy volunteers, the initial increase in heart rate and blood pressure was diminished. A decrease in sympathetic activity accompanied by an increase in parasympathetic activity could explain the tolerance that develops after repeated cannabis use [24]. Repeated marijuana use also causes the plasma volume to increase, which is hypothesized to result from increased aldosterone leading to sodium and water retention. This effect could be seen especially in the elderly exacerbating chronic illnesses such as congestive heart failure. Interestingly, tolerance to the cardiovascular effects of marijuana was lost as soon as 48 h after oral THC intake was stopped in one study [30]. In elderly patients who used marijuana as young adults, it is important to explain the rapid loss of tolerance that occurs with marijuana, as high doses of marijuana taken based on the belief of previous tolerance to the substance could prove to be disastrous.

## 6. Myocardial Infarction

Multiple case reports have linked marijuana to acute myocardial infarction (MI) over the past years [10,39,54,55]. Since then, numerous retrospective studies have been published to help understand that claim. In many instances, the patient is a young, healthy marijuana user who presents with chest pain and is found to have MI. These patients often do not have any cardiac risk factors, which leads the authors to conclude that marijuana is possibly the culprit. In the pediatric population, myocardial ischemia has also been reported with the use of synthetic cannabinoids (K2 and spice), which are starting to gain popularity [56].

In a study performed by Desai et al., the odds of developing MI reportedly increased by 8% in patients with recreational marijuana use [57]. Another study done by Ramphul et al. calculated an odds ratio of 5.03 (95% CI, 3.5–7.3; *p*-value < 0.01) for developing MI after cannabis use in teenagers [58]. Patients who admitted marijuana use had higher rates of out-of-hospital cardiac arrest during initial presentation [59]. The risk of developing MI in pediatric patients has been reported in numerous case reports. Thankavel et al. reviewed 32 cases of pediatric patients and found seven with vasospasms and MI associated with marijuana use [60].

Edible forms of marijuana have also been implicated in causing MI. In one case report, a 70-year-old man with known coronary artery disease presented with crushing chest pain, diaphoresis, and pallor after using a marijuana lollipop. The patient was subsequently diagnosed with non-ST elevation MI; nuclear medicine studies showed a worsening of his ejection fraction from 40% to 31% along with worsening in functional status [61]. Edible forms of marijuana often contain very high amounts of THC. Due to the erratic absorption of oral marijuana, the euphoric effects are delayed in most cases, leading to a higher THC amount being consumed and higher rates of complications [52].

A meta-analysis published in 2011 by Nawrot et al. investigating the triggers of non-fatal myocardial infarction found that cannabis smoking was the third-highest-ranking associated variable, with an odds ratio of 4.8 (95% CI, 2.9–9.5) [62]. Stroke and Transient Ischemic Attack (TIA) have also been reported, with one systemic review concluding that the risk of ischemic stroke was higher than that of other cardiovascular diseases among marijuana users [63]. The cardiovascular effects of marijuana have been observed in patients with HIV. In a study done by Lorenz et al., heavy marijuana users with HIV infection had a 19.7% rate of cardiovascular events compared to the 8.7% rate in occasional users and non-users (*p*-value < 0.012). This rate was significantly higher compared to that in HIV-infected men with no marijuana use, with heavy users having a 2.5-fold increased risk of developing cardiovascular events (95% CI, 1.2–5.3; *p*-value < 0.016) [64]. The increase in cardiac events was independent of tobacco use. A multitude of other case reports and studies in the literature with conflicting results have investigated the risk of cardiovascular (CV) events in marijuana users.

One of the hallmark studies that investigated marijuana as a trigger for MI was done by Mittleman et al. In this multicenter study, 3882 patients who had MI were interviewed within 4 days and data were collected for the determinants of MI Onset study (MIOS). The study used a case cross-over design. The results showed that the risk of myocardial infarction onset was elevated 4.8-fold (95% CI, 2.9–9.5; *p*-value < 0.001) within the first hour after smoking marijuana compared with that in periods of non-use [65]. In the second hour after smoking, the relative risk decreased to 1.7 (95% CI, 0.6–5.1; *p*-value < 0.34), which suggests a substantial decrease in cardiac effects in the second hour. The study also found a relative risk of 3.2 (95% CI, 1.4–7.3; *p*-value < 0.007) for smoking marijuana in the absence of other potential triggers of myocardial infarction. The mean age of users was 44 ± 8 compared to 62 ± 13 for non-users. Users were more likely to be men, active cigarette smokers, and obese. The study did not control for these factors due to a lack of power. In a systemic review of 46 papers, 14 cases showed that the time from last marijuana use to the onset of MI symptoms usually occurred within a 5 h window [41]. The timeline seems to suggest that while the risk of CV events may be decreased after the first hour of smoking marijuana, it is not diminished and can still cause significant events.

In patients with chronic stable angina, a study found that the anginal threshold was significantly decreased after smoking a single marijuana cigarette. Aronow and Cassidy showed that the exercise time to the onset of angina symptoms was decreased by an average of 48% after smoking a single marijuana cigarette, compared with a 8.6% decrease after smoking a marijuana placebo (a marijuana cigarette with no THC) [35]. The exercise time to angina symptoms had a 23% decrease after the smoking of a high-nicotine tobacco cigarette, much lower than that after marijuana smoking. The authors attributed the results to an increase in carboxyhemoglobin levels leading to ischemia, hemodynamic changes causing plaque rupture, and endothelial damage leading to cell death.

The mechanism behind marijuana-induced MI is unknown. One hypothesis from Stanley and O’Sullivan is that cannabis induces transient coronary vasospasm [66]. This hypothesis is supported by many case reports of patients presenting with MI and normal coronary vessels upon cardiac angiography [67]. Another hypothesis is that the myocardial oxygen supply–demand mismatch causes transient ischemia. Oxygen supply is decreased due to an increase in carboxyhemoglobin levels following marijuana smoking. Additionally, marijuana smoking is known to cause an increase in myocardial oxygen demand [68]. Marijuana is found in some studies to contribute to endothelial dysfunction, a key mechanism in atherosclerosis. In one study, marijuana users were noted to have abnormal flow-mediated dilation in the arterial bed, which is indicative of endothelial dysfunction [41].

Thrombus formation has also been reported in cases of marijuana-induced MI. In a case report published by Wengrofsky et al., a case of a 30-year-old African American male with no cardiovascular disease (CVD) risk factors who presented with recurrent ST-Elevation Myocardial Infarction (STEMI) was reported. Coronary angiography showed recurrent 100% occlusion of the left anterior descending (LAD) artery with no stenosis and a calcium score of zero. The authors hypothesized that marijuana-associated thrombosis is responsible for his recurrent occlusion. Other potential mechanisms include CB1R-mediated endothelial dysfunction and vasospasms leading to total arterial occlusion resulting in ischemia [69]. THC-induced endothelial dysfunction is caused by oxidative stress and reduced nitric oxide production. These events lead to the enhanced activation of factor seven, further contributing to platelet aggregation and thrombus formation in the coronary arteries [41]. Plaque rupture due to hemodynamic stress as a result of smoking marijuana has also been suggested as a possible mechanism. These patho-physiologic effects are likely mechanisms for MI in some marijuana users.

Electrocardiogram (EKG) findings in marijuana users presenting with MI often show ST segment elevation, which was documented in 60% of EKGs in one study. The most common angiographic finding was LAD coronary artery occlusion in 35% of patients. Concomitant cardiomyopathy was described in 34% of cases [67]. In a systemic review of 46 articles, angiographic findings in 36.8% of patients revealed normal coronaries [41]. In another prospective, cross-sectional study of 138 patients of whom 23 were cannabis users, it was reported that users had increased proportions of ST elevation MI, cardiomyopathy, and coronary artery disease compared to non-users [54].

Morality following MI also seems to be affected by marijuana use. Mukamal et al. conducted an inception cohort study that revealed marijuana to be associated with a three-fold higher mortality rate after MI. They also showed a graded increase in the risk of MI with more frequent marijuana use, citing less than weekly users having a hazard ratio of 2.5 (95% CI, 0.9–7.3) and weekly marijuana users having a hazard ratio of 4.2 (95% CI, 1.2–14.3) [70]. These results were further confirmed by Frost et al., who found that the mortality rate was 29% higher among cannabis users with MI compared to that among non-users [71]. The results of the National Health and Nutrition Examination Survey found marijuana use to be significantly associated with increased hypertension mortality from myocardial infarction and stroke [67]. In a study that investigated the long-term outcomes of marijuana use in adults with their first MI at <50 years of age, the results showed that marijuana use is associated with increased all-cause mortality, with a hazard ratio of 1.47 (95% CI, 0.90–2.42; *p*-value < 0.012). After adjusting for age and different chronic conditions, marijuana users had increased cardiovascular mortality, with a hazard ratio of 2.13 (95% CI, 1.03–4.42; *p*-value < 0.042). Additionally, the authors noted that the marijuana users had a higher rate of out-of-hospital cardiac arrest associated with MI [59].

On the other hand, in recent years, there have been multiple studies that have reported a lack of association between marijuana use and cardiovascular disease. The Coronary Artery Risk Development in Young Adults (CARDIA) study is a longitudinal, multicenter, cohort study that examined the development of cardiovascular disease in young men aged 18–30 years old. The study population consisted of young, healthy adults who were followed for 25 years. The results showed no evidence to suggest that cumulative lifetime or recent marijuana use changes the risk of future cardiovascular events in middle age. One explanation for these results could be the low lifetime dose of marijuana in the study participants. The typical participant smoked less than one cigarette per day and quit in early adulthood, which is not the behavior that we currently observe in marijuana users [72]. Quantifying the dose and frequency of marijuana use continue to pose a great challenge and often complicates the comparison of different studies.

## 7. Cardiac Arrythmias

Cardiac arrythmias and palpitations have been reported in the setting of marijuana use, with the incidence of arrythmias increasing by two-fold in users [13]. Heavy marijuana users have a greater risk of developing palpitations compared to lighter users, with daily users having a relative risk of 2.2 (95% CI, 1.6–3.3; *p*-value < 0.0001) compared to a relative risk of 1.4 (95% CI, 1.1–1.9; *p*-value < 0.006) in occasional users [73]. A retrospective review of nearly 2.5 million patients in the National Inpatient Sample database found that 2.7% of marijuana users experienced arrythmias, with atrial fibrillation being the most common subtype [74]. Korantzopoulos et al. reported an average age of 24 years old for patients experiencing cannabis-induced arrythmias, with all cases experiencing atrial fibrillation shortly after smoking marijuana [75]. The most common arrythmia found in a study done by Kariyanna et al. was atrial fibrillation (26%), followed by ventricular fibrillation (22%). A Brugada pattern was also reported in 19%; the inhibitory effects of THC on sodium and potassium channels may explain Brugada pattern that is induced by marijuana use [76]. The study also reports death occurring in three cases due to ventricular fibrillation and sudden cardiac death. Additionally, the all-cause hospital mortality of cannabis users with arrythmias increased from 3.7% to 4.4% from the year 2010 to 2014 [13].

The rhythm disturbances reported with marijuana include sinus tachycardia, ectopic atrial or ventricular rhythm, and atrial or ventricular fibrillation. Most authors attribute these tachyarrhythmias to a hyperadrenergic state after marijuana use [6]. Adrenergic stimulation causes a reduction in action potential duration and results in a microreentrant tachycardia [68]. It has been reported that marijuana might be the sole precipitating factor for atrial fibrillation in individuals younger than 45 years old with a recurrence of atrial fibrillation occurring repeatedly after every exposure [6]. For patients with difficult-to-control atrial fibrillation, inquiring about marijuana use might prove to be more beneficial than other methods aiming to control it.

Marijuana use has also been implicated in premature ventricular contractions (PVCs), *p* and T wave changes, and reversible ST segment changes [68]. The authors in a study done by Beaconsfield et al. had volunteers who smoked marijuana placed on simultaneous EKG monitoring systems, which showed a decreased amplitude of the *p* waves following consumption, suggesting an atrial abnormality [21]. Miller et al. later studied the effects of THC administration on patients and found the development of frequent PVCs, which disappeared in 15 min after stopping the THC infusion. They concluded that THC decreases the sinoatrial (SA) conduction time and atrioventricular (AV) conduction time, after also noting a decrease in the AV nodal refractory period [77]. The reduction in the atrial refractory period could result in a reentrant mechanism of atrial fibrillation [68]. In a case report, Khouzam et al. postulated that PVCs could be related to a slow coronary flow phenomenon in a marijuana user. In these cases, counseling the patients to abstain is the goal to control PVCs and subsequently prevent recurrent tachyarrhythmias [78].

Cases of ventricular arrythmias have also been reported, which are hypothesized to be secondary to excessive catecholamine release [68]. In an autopsy study done by Bachs et al., they found THC to be the only drug present in the postmortem serum samples of six young adults with ages ranging from 17 to 43 years old who presented with sudden death shortly after marijuana use [79]. In another case report by Rezkalla et al., a 34-year-old man who presented with syncope after marijuana use was found to have ventricular tachycardia and a significant reduction in coronary blood flow. After placing the patient on verapamil and the cessation of marijuana use, coronary blood flow normalized and the tachycardia was no longer inducible. The authors attributed the results to marijuana impacting the activity of the Purkinje fibers by altering the coronary microcirculation [80]. The mechanism of ventricular arrythmia induction by THC is unclear; however, it is possibly related to the sympathetic stimulation following marijuana use resulting in tachycardia.

## 8. Heart Failure and Cardiomyopathy

A link between marijuana use and heart failure (HF) development or exacerbation has not been firmly established. In a recent cross-sectional, retrospective review of the database of hospital discharge diagnosis, marijuana use was found to be an independent predictor of heart failure, with an odds ratio of 1.1 (95% CI, 1.03–1.18; *p*-value < 0.01). The prevalence of hypertension tends to be significantly greater in marijuana users than in non-users; however, the direct impact of marijuana on hypertensive heart disease is not well known and may need further investigation [9]. Similarly, marijuana use leads to myocardial supply and demand mismatch, but its long term impact on ischemic cardiomyopathy is not well described.

Takotsubo is perhaps the most reported cardiomyopathy in marijuana users. In a multivariate, binary analysis from a study of 33,343 patients, active marijuana use was noted to double the risk of Takotsubo cardiomyopathy in young men. This study included patients who were hospitalized with stress cardiomyopathy between 2003 and 2011 in the U.S., less than 1% of whom were marijuana users. Marijuana users were more likely to develop cardiac arrest (2.4% vs. 0.8%, *p*-value < 0.034) and to require an implanted defibrillator (2.4% vs. 0.6%, *p*-value < 0.008). Cannabis use was found to be an independent predictor of transient ventricular regional ballooning, a marker of Takotsubo cardiomyopathy. The authors concluded that marijuana users had increased morbidity compared to non-users [81]. There is a lack of studies investigating the pathophysiology of marijuana use and the development of Takotsubo cardiomyopathy.

Interestingly, Takotsubo cardiomyopathy has been reported with concomitant marijuana hyperemesis syndrome. One case report described a young, healthy patient who presented with marijuana hyperemesis symptoms, nonspecific T wave changes, and mild troponin elevation. Echocardiogram findings showed severe mid-ventricular hypokinesis and left ventricular ejection fraction in the range of 30–35%, consistent with a variant of Takotsubo cardiomyopathy. Cardiac angiography showed normal coronaries. The authors attributed the symptoms to the hypercatecholaminergic state that resulted from marijuana ingestion [82]. It is not clear if patients who develop marijuana hyperemesis syndrome are at an increased risk for developing Takotsubo cardiomyopathy compared to other marijuana users. Another case report by Del Buono et al. reported a 23-year-old female with a history of chronic marijuana use who presented with ventricular fibrillation/cardiac arrest and was noted to have mid-wall Takotsubo cardiomyopathy [83]. An echocardiogram showed severe left ventricular systolic dysfunction with a left ventricular ejection fraction in the range of 20–25%. Coronary angiography showed normal coronaries and a left ventriculogram showed mid-segment akinesia with the sparing of the base and the apex, consistent with the mid-wall variant of Takotsubo cardiomyopathy. The patient had a modest improvement in the left ventricular function during her hospitalization; however, she passed away due to anoxic brain injury [67].

## 9. Cannabis Arteritis

Cannabis arteritis has been described in multiple cases in the literature and is mostly associated with high-dose cannabinoids. The first cases described in 1960 involved 29 Moroccan cannabis smokers who developed obliterative arteritis [84]. Since then, more than 100 case reports of cannabis arteritis have been published in the literature. Most reports have been of young men who developed unilateral digital necrosis and ulcers of the lower limbs with occasional occurrences of thrombophlebitis and Raynaud’s phenomenon [85]. Interestingly, patients with cannabis arteritis have an early disappearance of pedal pulses with most case reports citing nonpalpable pulses upon presentation [86,87]. Upper limb involvement is less frequently reported [85]. Cannabis arteritis has a poor prognosis without cessation of the drug, with more than 50% of patients undergoing limb amputation due to worsening disease [85].

Tissue necrosis and gangrene seem to worsen during periods of heavy marijuana use, with remission noted after stopping use [30]. In a case report by Combemale et al., they describe a tobacco smoker with heavy cannabis use who presented with painful necrosis of his toes [88]. He had worsening flares during heavy cannabis use, while his tobacco use remained steady. Cannabis arteritis is very similar to Buerger’s disease. Most cannabis arteritis cases have been reported in patients with concomitant tobacco use, raising the question of whether tobacco is the main substance behind the disease. However, smoking cessation often does not impact disease progression, which refutes a diagnosis of Buerger’s disease where it is expected that smoking cessation leads to an improvement of symptoms [86]. Patients in both conditions are often young men who present with claudication and painful distal ischemia. Angiograms show circulatory abnormalities very similar to those of Buerger’s disease, with distal segmental narrowing of the arteries [89]. In contrast to Buerger’s disease, the collateral arterial network is less developed in cannabis arteritis and proximal atheromatous lesions are sometimes seen [87]. An arterial biopsy in one case report found thrombosis with endarteritis associated with inflammation and fragmentation of the internal elastic lamina [89].

The pathophysiology of cannabis-associated arteritis is still under debate. Vasoconstriction due to THC is often cited as the mechanism leading to cannabis arteritis [86]. Another hypothesis regarding the development of cannabis arteritis concerns arsenic as a contaminant. Arsenic impairs angiogenesis by inhibiting vascular endothelial growth factor and inducing endothelial cell apoptosis [88]. The rare cases of cannabis arteritis could be associated with marijuana containing higher amounts of arsenic. Migratory thrombophlebitis has also been reported as a possible complication of cannabis use [6]. The pro-coagulant effects of marijuana and platelet activation could explain these findings.

## 10. Discussion

Cannabis is an incredibly versatile compound, with over 500 chemicals sometimes involved [90]. While previous studies were focused on the effects of THC on cardiovascular health, new research is investigating the effects of CBD and other compounds in cannabis. The potency of marijuana is an important variable in the available research studies. In the U.S., one author reports that while the THC levels available for researchers average about 12%, THC levels in the market sometimes exceeds 35% [91]. While it is currently impossible to ascribe marijuana use as the sole participant in the various cardiovascular events described, strong evidence exists suggesting a link between marijuana use and cardiovascular events. Pathophysiological explanations for myocardial infarction induction by marijuana often attribute it to vasospasms and oxygen supply/demand mismatch. The hyperadrenergic state and oxidative stress created by cannabis have also been linked to the development of arrythmias and endothelial damage. New research is currently focused on understanding the inflammatory environment that could be related to cannabis use and the possible therapeutic benefits of some cannabis compounds that have proven to be anti-inflammatory. Important information about marijuana and atherosclerosis development and marijuana’s impact on cell signaling is also starting to emerge. Information about the frequency, route, dose, and cumulative lifetime exposure is often missing in research studies, which creates challenges when comparing their results. With mounting evidence linking cardiovascular events to marijuana use, it is extremely important to stay committed to conducting quality research on this topic, both basic science studies and clinical trials.

Marijuana use has become extremely prevalent in our society, which necessitates that we stay informed and also involve our patients in a risk/benefit discussion concerning its use. In patients with established cardiovascular risk factors and cardiovascular disease, we recommend an open conversation concerning known risks linked to marijuana use. We also recommend involving the expertise of a clinical pharmacist to evaluate drug interactions and making adjustments to the patient’s medications accordingly. A urine toxicology screen for young patients presenting with MI, new onset arrythmia, difficult-to-control atrial fibrillation, or Takotsubo cardiomyopathy could provide diagnostic clues and aid in management. We recommend inquiring about marijuana use regularly, especially focusing on the frequency, method of use, and potency if known. Concomitant drug use should also be discussed with these patients, as it has been previously reported that cannabis use is associated with increased tobacco and alcohol use [90]. The emerging issue of unregulated cannabis products with high potency and unknown consequences should not be ignored. State and federal laws often present barriers when conducting marijuana-related research, which can further limit the scientific community’s knowledge in this area [92]. Large prospective studies are needed to assess the link between cardiovascular events and marijuana use.

## 11. Conclusions

Marijuana use has increased tremendously over the past decade, with new forms and differing potencies available on the market. Although it is widely viewed as a safe drug, marijuana has been strongly linked to various cardiovascular adverse events over the years. Many cases have linked marijuana to myocardial infarction, especially in young healthy men with no other risk factors. Marijuana has also been associated with a worse mortality rate post MI. Cases of marijuana precipitating arrythmias, stress cardiomyopathy, and arteritis have all been described. With the rise in cannabis use among older patients, who are the most vulnerable to cardiovascular events, it is expected that these reports will increase in the next few years. The pathophysiology of these events is still debated, with contradictory studies available in the literature. The expansive presence of the endocannabinoid receptors in the human body makes drawing conclusions extremely challenging. The interactions of the endocannabinoid system with the autonomic nervous system seem to be the driving force behind the reported cardiovascular adverse events. The lack of laboratory studies regarding the impact of marijuana on human cardiac physiology makes interpreting case reports very difficult. However, the alarming rate of adverse cardiovascular events reported over the past decade necessitates that physicians remain vigilant in everyday practice to recognize these effects and counsel their patients accordingly.
